# The relation of whole grain surrogate estimates and food definition to total whole grain intake in the Finnish adult population

**DOI:** 10.1007/s00394-023-03119-7

**Published:** 2023-02-27

**Authors:** Rilla Tammi, Satu Männistö, Heli Reinivuo, Heli Tapanainen, Jenna Rautanen, Niina E. Kaartinen

**Affiliations:** Department of Public Health and Welfare, Finnish Institute for Health and Welfare, P.O. Box 30, 00271 Helsinki, Finland

**Keywords:** Cereals, Fiber, FinHealth study, Food composition database, Rye, Whole grain

## Abstract

**Objectives:**

Challenges in estimating total whole grain intake have led to the use of surrogate estimates, of which accuracy has not been assessed. We examined the suitability of five potential surrogates (dietary fiber; bread; rye bread; rye, oat and barley combined; rye) and a whole grain food definition to measure total whole grain intake in the Finnish adult population.

**Methods:**

Our data comprised 5094 Finnish adults participating in the national FinHealth 2017 Study. Dietary intake was assessed by a validated FFQ. Food and nutrient intakes, including total whole grain, were calculated utilizing the Finnish Food Composition Database. The Healthgrain Forum whole grain food definition was applied to examine definition-based whole grain intake. Spearman correlations and quintile cross-classifications were calculated.

**Results:**

Definition-based whole grain intake and consumption of rye, oat and barley combined had consistently the strongest correspondence with total whole grain intake. Rye and rye bread consumption also corresponded well with total whole grain intake. The correspondences of dietary fiber and bread with total whole grain were lower and more affected by the exclusion of energy under-reporters. Furthermore, their correlations with total whole grain intake varied the most between population subgroups.

**Conclusions:**

Rye-based estimates, especially rye, oat and barley combined, and definition-based whole grain intake appeared suitable surrogate estimates of total whole grain intake for epidemiological research of Finnish adults. The variation between surrogate estimates in their correspondence with total whole grain intake demonstrated the need for further evaluation of their accuracy in different populations and regarding specific health outcomes.

**Supplementary Information:**

The online version contains supplementary material available at 10.1007/s00394-023-03119-7.

## Introduction

Whole grain intake has been suggested to be beneficial in the prevention of several lifestyle-related chronic diseases, such as cardiovascular diseases and certain cancers [1–4]. Furthermore, whole grains have been deemed a key component in diets that promote environmental sustainability [5]. Yet, whole grain research is currently limited by methodological discrepancies that impede comparability between studies and may attenuate the found associations between whole grain intake and health outcomes. These limitations include the estimation of whole grain intake based on surrogate estimates, of which correspondence to total whole grain intake has not been studied. In addition, differences in whole grain definitions contribute to the discrepancies.

Whole grains are defined based on their processing rather than chemical composition. Thus, in addition to nutritional knowledge, quantifying whole grain content in foods requires detailed information on the current food processing practices that are used in the production of cereal-based foods. As collecting this information is laborious and time-consuming, comprehensive information on whole grain content in foods is commonly lacking from food composition databases. Furthermore, as the food processing practices differ between countries, information on whole grain composition collected in one country may not be directly applicable elsewhere. Consequently, utilizing other whole grain-related variables that already exist in food composition databases (e.g., fiber) or can be more easily measured or derived from dietary data with less precision at the nutrient level (e.g., consumption of whole grain-containing foods) may often be the most feasible way to estimate whole grain intake.

Several surrogate estimates have been applied worldwide to approximate whole grain intake instead of calculating total whole grain intake from the whole diet as recommended [6]. The estimates have mainly been based on the consumption of certain whole grain-containing foods, such as whole grain bread or breakfast cereals [7–11]. However, the accuracy of these estimates in relation to total whole grain intake has not been assessed.

Intake of dietary fiber could as well be considered a potential surrogate estimate for total whole grain intake. In the US National Health and Nutrition Examination Survey (NHANES) 2009–2010, adults following the recommendation of at least three ounce equivalents (48 g) of whole grains per day were more likely to be in the highest tertile of fiber intake than others [12]. Moreover, whole grain intake has been repeatedly associated with higher fiber intake compared to non-consumers [13–17]. However, while whole grains are predominantly high in fiber, fibers can originate from various food sources, such as vegetables, fruits and refined grains. Thus far, the suitability of dietary fiber to estimate total whole grain intake has not been evaluated.

In addition to surrogate estimates, whole grain intake has been assessed based on the consumption of whole grain foods defined by varying cut-off values (e.g., ≥ 10%, ≥ 51%) for whole grain content [7, 13, 18–21]. So far, the correspondence of cut-off-based estimates with total whole grain intake has been examined in few studies. In two British studies, applying a ≥ 51% cut-off for whole grain content significantly underestimated whole grain intake in the adult population in comparison with total whole grain intake or intake of foods with ≥ 10% whole grain content [13, 19]. This was also apparent in a recent study in the Australian population in which the Healthgrain Forum’s whole grain food definition (foods containing ≥ 30% whole grains and more whole than refined grains) was applied in comparison with total whole grain intake [21]. Aside from the quantity of whole grains, whole grain food definitions consider whole grain sources and their healthiness (e.g., Healthgrain Forum definition). Hence, utilizing a whole grain food definition can be useful, for example, in research where the complex interrelationships between food components need to be considered. Yet, research is required to elucidate how well findings derived by applying a whole grain food definition represent total whole grain intake and how comparable these values are.

As the challenges in calculating whole grain content in foods remain and whole grain data is frequently lacking from food composition databases, surrogate estimates for total whole grain intake will be required also in the future. Thus, better understanding of the surrogates is warranted to facilitate whole grain intake estimation when total whole grain intake from the whole diet cannot be directly derived and to standardize the estimation between studies. More coherent estimation methods will, in turn, benefit the consistency of results regarding the associations between whole grain intake and health outcomes. The need for more harmonized whole grain research is underlined by whole grains’ focal role in sustainable diets, toward which the global population should increasingly shift in the future [5].

Our objective was to assess the suitability of five potential surrogate estimates (dietary fiber; bread; rye bread; rye, oat and barley combined; rye) in the estimation of total whole grain intake in the Finnish adult population and population subgroups (by sex, age, education and body mass index (BMI)) in the context of epidemiological research. Furthermore, we examined the correspondence between total whole grain intake and whole grain intake estimated based on the Healthgrain Forum whole grain food definition.

## Methods

### Study population

We applied data from the population-based FinHealth 2017 Study examining the health, well-being, health behavior and functional capacity of the Finnish adult population [22]. The study was conducted in individuals aged 18 years and older residing in mainland Finland. The data were gathered between January and May 2017, including self-administered questionnaires and a health examination. An invitation to participate in the health examination was sent via mail to 10 247 individuals randomly chosen by two-staged cluster sampling from the national population register. One self-administered questionnaire was sent along with the invitation letter, while the remaining questionnaires were distributed in the health examinations to be filled in either during the examination visit or at home and mailed to the Finnish Institute for Health and Welfare. All questionnaires could be completed either on paper or electronically. Our data consist of individuals who participated in the health examination (58% of the invited) and completed the food frequency questionnaire (FFQ) acceptably. Those with incompletely filled FFQs (*n* = 110), duplicate answers (*n* = 9, paper and electronic FFQ), and consent withdrawal (*n* = 7) were excluded. In addition, participants pregnant (*n* = 31) or in the 0.5% extremes of the sex-specific daily energy intake distributions were excluded (*n* = 51). Thus, our final data comprised 2844 women and 2250 men.

Written informed consent was obtained from all participants and the study was conducted according to the guidelines laid down in the Declaration of Helsinki. All procedures involving human subjects were approved by the Coordinating Ethics Committee at the Hospital District of Helsinki and Uusimaa.

### Diet

Dietary intake was assessed by a validated, semi-quantitative, 134-item FFQ inquiring into habitual food consumption over the past 12 months [23–25]. Participants received the FFQ in the health examination along with oral and written instructions for its self-administered completion. The questionnaire items included foods, mixed dishes and beverages. Participants reported their consumption of each item according to ten frequency categories ranging from none to six or more times a day. The quantity of consumption was estimated in fixed, sex-specific portion sizes (e.g., slice, glass, volume). Each item in the questionnaire was composed of 1–9 foods commonly consumed in the Finnish adult population based on the national FinDiet 2017 Survey [22, 26]. Each of these foods (altogether 451 foods, Supplementary Table S1) encoded a food in the Finnish Food Composition Database Fineli^®^ [27]. Alongside Fineli^®^, in-house software was applied to calculate the average daily intake of foods (g/d), nutrients and energy (MJ/d). To calculate the consumption of basic ingredients and further the intake of nutrients and energy, the foods within the FFQ items were broken down into basic ingredients (ingredients that are not broken down further in the database) based on their standard recipes. In our study, three calculation levels were used to derive the examined whole grain intake surrogate estimates: (1) food consumption (consumption of total and rye bread), (2) basic ingredient consumption (consumption of rye, oat, and barley), (3) nutrient and energy intake (intake of dietary fiber and total whole grain).

### Estimating whole grain intake

A new whole grain database was compiled within Fineli^®^ to calculate total whole grain intake. Whole grains were defined according to the Healthgrain Forum as the intact, ground, cracked or flaked kernel after the removal of inedible parts, in which the principal anatomical components (endosperm, germ and bran) are present in the same relative proportions as in the intact kernel [28]. Small losses of components in the processing are allowed. The definition includes grains from the *Poaceae* family, such as wheat, rye, oat, barley, rice, maize, sorghum and millet. Furthermore, pseudocereals, such as amaranth, buckwheat and quinoa, are included.

Total whole grain composition was first assigned by hand for each basic ingredient in the database. Basic ingredients include, for example, flour, flakes, cereal mixtures, rice and popcorn, as well as certain foods, such as oat and rice-based drinks, muesli bars and pasta. The assigned whole grain values for the basic ingredients were then utilized to calculate whole grain content in foods based on the proportions of basic ingredients in their standard recipes. The calculations were done by in-house software [27], and all values were manually verified. Product labels and information from manufacturers were utilized when further verification was required (also on foods categorized as basic ingredients). Whole grain values were assigned as grams in dry weight per 100 g [6].

We examined five dietary variables as potential surrogate estimates for total whole grain intake. These included dietary fiber intake, and consumption of bread, rye bread, rye, and a combination of rye, oat and barley. Dietary fiber and bread represent variables best applicable across Western food cultures. Bread included the following three FFQ items: rye bread; multigrain bread, graham roll or toast; and French bread, baguette or other white wheat bread. Rye bread and rye were chosen for the examination due to their focal role in the Finnish food culture. In addition to rye, both oat and barley are primarily consumed as whole grain in Finland. Thus, combining rye, oat and barley was hypothesized to derive high correspondence with total whole grain intake.

We also estimated whole grain intake only including foods that contain at least 30% whole grain ingredients and more whole than refined grains on a dry-weight basis, according to the whole grain food definition of the Healthgrain Forum [29]. Overall, 16 of the 134 items in the FFQ included at least one food with whole grain ingredients (Supplementary Table S2). All whole grain-containing foods within nine of the 16 FFQ items fulfilled the Healthgrain Forum definition criteria. These nine items are specified in Supplementary Table S2. Within the item ‘Multigrain bread, graham roll or toast’, only one food fulfilled the criteria. As the contribution of this food for the FFQ item was very small, it was excluded to facilitate the calculation process.

### Sociodemographic factors and anthropometric measures

Information on participants’ sex and age was derived from the sampling frame. Educational level was inquired about in a self-administered questionnaire as the total number of school years. Based on this information, participants were classified into educational tertiles (low, medium, high) by sex and birth year to consider the extension of the basic education system and the increase in average school years over time. Height (cm) and weight (kg) were measured in the health examinations according to international standard protocols by trained research staff [30, 31]. BMI (kg/m^2^) was calculated by dividing weight (kg) by squared height (m).

### Statistical analyses

Means and standard deviations for age and intake of energy, foods and nutrients, as well as proportions for participants with high education, participants with obesity (BMI ≥ 30 kg/m^2^) and energy under-reporters, were calculated by sex and in total population. Energy under-reporters were identified by calculating the ratio between energy intake (EI) and basal metabolic rate (BMR) [32] and applying 1.14 as a cut-off, a smaller value representing under-reporting (EI:BMR ≤ 1.14) [33]. Spearman’s rank correlation coefficients were calculated between total whole grain intake and whole grain surrogate estimates (dietary fiber; bread; rye bread; rye, oat and barley combined; rye), as well as whole grain food definition-based whole grain intake. Spearman’s rank correlation was applied as the distribution of rye bread and rye consumption remained skewed despite log transformation. Partial Spearman’s correlations controlled for energy intake were calculated in the total population and subgroups (sex, age, education, BMI) and after the exclusion of energy under-reporters. Correlations r > 0.7 were considered strong. For the subgroup analyses, binomial age, education and BMI variables were created. Age was categorized according to its median value (58 years), low and medium education groups were combined oppose to the high education group, and a cut-off-value of 30 kg/m^2^ was applied for BMI to classify participants either into those with normal weight and overweight (BMI < 30 kg/m^2^) or those with obesity (BMI ≥ 30 kg/m^2^). P-values for the differences in correlations between subgroups were calculated using Fisher Z scores. A two-tailed *p* value of < 0.05 was considered statistically significant. The correspondence of the ranking of participants between total whole grain intake and whole grain surrogate estimates or whole grain food definition-based whole grain intake was examined by cross-classification of intake quintiles. For this, all examined variables, including total whole grain intake, were categorized into quintiles. The proportions of participants categorized into the same or adjacent quintile or the opposite quintile (first versus fifth quintile, gross misclassification) between total whole grain intake and whole grain surrogate estimates or definition-based whole grain intake were calculated. In addition to the unadjusted values, cross-classification was conducted with energy-adjusted values (g/MJ), as well as excluding energy under-reporters from the unadjusted analysis. All statistical analyses were performed by IBM SPSS Statistics (version 27; IBM Corp.).

## Results

The participants were on average 56 years old (age range 19 − 99 years), and one fourth were living with obesity both in women and men (Table 1). Daily total whole grain intake was 56 g (7.0 g/MJ) in women and 65 g (6.6 g/MJ) in men. When whole grain food definition was applied, whole grain intake tended to be slightly lower than total whole grain intake (52 g/d in women, 60 g/d in men). The daily intake of dietary fiber was 22 g in both sexes. The consumption of bread was 82 g/d in women and 91 g/d in men. More than half of the bread consumption was covered by rye bread (53 g/d in women, 62 g/d in men). Daily intake of rye, oat and barley combined was 57 g in women and 64 g in men, while the intake of rye alone was 41 g/d in women and 46 g/d in men.

**Table 1:** Characteristics of participants and daily intakes of energy and whole grain-related dietary variables (mean values and standard deviations (SD) or percentages) in the FinHealth 2017 Study

	Women (*n* = 2844)		Men (*n* = 2250)		Total (*n* = 5094)	
	Mean/%	SD	Mean/%	SD	Mean/%	SD
Characteristics						
Age (years)	56	16	56	16	56	16
Participants with high education^a^ (%)	34		37		35	
Participants with BMI ≥ 30 kg/m^2^ (%)	25		25		25	
Energy under-reporters^﻿b^ (%)	36		41		38	
Dietary intake						
Energy (MJ)	7.9	2.6	9.7	3.3	8.7	3.1
Total whole grain^c^ (g)	56	36	65	45	60	40
Total whole grain^c^ (g/MJ)	7.0	3.9	6.6	3.9	6.9	3.9
Whole grain (whole grain food definition)^d^ (g)	52	35	60	44	55	39
Dietary fiber (g)	22	10	22	10	22	10
Bread (g)	82	55	91	63	86	59
Rye bread (g)	53	45	62	52	57	49
Rye, oat and barley combined (g)	57	39	64	46	60	42
Rye (g)	41	33	46	38	43	35

*BMI* Body Mass Index

^a^Participants were classified into educational tertiles based on self-reported total number of school years, and by sex and birth year to consider the extension of the basic education system and increase in school years over time

^b^Energy under-reporters were identified as participants with the ratio between energy intake and basal metabolic rate ≤ 1.14 (Goldberg cut-off value) [32, 33]

^c^Total whole grain intake was calculated on a dry weight-basis including all whole grains from the diet

^d^Whole grain intake from foods with ≥ 30% whole grain ingredients and more whole than refined grains on a dry-weight basis [29]

Total whole grain intake correlated strongly with the intakes of all surrogate estimates as well as whole grain food definition-based whole grain intake, the Spearman’s rank correlation coefficient ranging from 0.77 for bread consumption to 0.99 for intakes of whole grain (whole grain food definition) and the combination of rye, oat and barley (Table 2). The correlations remained similar after controlling for energy intake apart from dietary fiber intake and bread consumption, for which the correlations decreased from 0.78 and 0.77 to 0.68 and 0.69, respectively. The exclusion of energy under-reporters did not change the results markedly. Approximately 40% of the participants were identified as energy under-reporters. Examining the intakes as g/MJ instead of controlling for energy intake derived very similar results as well (data not shown).

**Table 2:** Correlation of daily total whole grain intake with whole grain food definition-based whole grain intake and intake of five potential whole grain surrogate estimates

Dietary intake (g/d)	Total whole grain intake (g/d)		
	r^a^	r^b^	r^b,c^
Whole grain (whole grain food definition)^d^	0.99	0.99	0.99
Dietary fiber	0.78	0.68	0.67
Bread	0.77	0.69	0.68
Rye bread	0.86	0.84	0.85
Rye, oat and barley combined	0.99	0.99	0.99
Rye	0.88	0.85	0.86

^a^Spearman’s rank correlation coefficient

^b^Partial Spearman’s rank correlation coefficient controlled for energy intake

^c^Energy under-reporters excluded. Energy under-reporters were identified as participants with the ratio between energy intake and

basal metabolic rate ≤ 1.14 (Goldberg cut-off value) [32, 33]

^d^Whole grain intake from foods with ≥ 30% whole grain ingredients and more whole than refined grains on a dry-weight basis [29]

In the population subgroup analyses, statistically significant differences in the partial Spearman’s correlations controlled for energy intake occurred between each of the examined subgroups (sex, age, education and BMI) (Table 3). The significant differences indicated, in general, stronger correlations between total whole grain intake and the examined variables in men than women, participants aged ≥ 58 years than younger, and in participants with obesity than those living with normal weight or overweight. Albeit statistically significant, the differences were mainly small. Greater differences appeared between women and men in the correlations for dietary fiber intake and bread consumption. Regarding dietary fiber, a strong correlation (*r* = 0.75) occurred in men, while in women, the correlation was 0.64 (*P* < 0.0001). The correlation between total whole grain intake and bread consumption was 0.65 in women and 0.73 in men (*P* < 0.0001). These differences, along with stronger correlations for the combined consumption of rye, oat and barley in men than women and for bread consumption in participants with obesity than those with normal weight or overweight, remained significant when energy under-reporters were excluded from the analyses (Supplementary Table S3). Moreover, the results were similar when g/MJ values were applied (data not shown).

**Table 3:** Correlation of daily total whole grain intake with whole grain food definition-based whole grain intake and intake of five potential whole grain surrogate estimates by sex, age, education and body mass index (BMI)

Dietary intake (g/d)	Total whole grain intake (g/d)											
	Women	Men		Age < 58^a^	Age ≥ 58^a^		Education low/medium^b^	Education high^b^		BMI < 30 kg/m^2^	BMI ≥ 30 kg/m^2^	
	r^c^		*P*^d^	r^c^		*P*^d^	r^c^		*P*^d^	r^c^		*P*^d^
Whole grain (whole grain food definition)^e^	0.99	0.99	0.03	0.99	0.99	< 0.001	0.99	0.99	0.32	0.99	0.99	0.50
Dietary fiber	0.64	0.75	< 0.0001	0.65	0.66	0.42	0.70	0.66	0.01	0.68	0.71	0.05
Bread	0.65	0.73	< 0.0001	0.67	0.65	0.25	0.69	0.67	0.09	0.67	0.74	< 0.0001
Rye bread	0.84	0.83	0.22	0.81	0.83	0.03	0.83	0.84	0.28	0.83	0.86	< 0.01
Rye, oat and barley combined	0.99	0.99	< 0.0001	0.99	0.99	0.10	0.99	0.99	0.20	0.99	0.99	0.43
Rye	0.86	0.85	0.28	0.83	0.84	0.04	0.85	0.86	0.33	0.84	0.88	< 0.001

^a^Categorized according to the median age

^b^Participants were classified into educational tertiles based on self-reported total number of school years and by sex and birth year to consider the extension of the basic education system and increase in school years over time

^c^Partial Spearman’s rank correlation coefficient controlled for energy intake

^d^Differences between independent sample correlation coefficients were tested using Fisher Z scores

^e^Whole grain intake from foods with ≥ 30% whole grain ingredients and more whole than refined grains on a dry-weight basis [29]

The proportion of participants categorized into the same quintile according to the intake of total whole grain and whole grain surrogate estimates or definition-based whole grain intake (unadjusted) ranged from approximately 50% for dietary fiber intake and consumption of bread, rye bread and rye to 88% for the combination of rye, oat and barley and 89% for definition-based whole grain intake (Table 4). For each examined variable, the proportion categorized into the same or adjacent quintile was greater than 85% and close to 100% for the definition-based whole grain intake and consumption of rye and rye, oat and barley combined. Gross misclassification was rare and only occurred with dietary fiber intake (2%) and bread consumption (4%). However, when energy under-reporters were excluded from the analysis, the proportion of grossly misclassified participants, categorized into the lowest quintile of total whole grain intake and highest quintile of dietary fiber intake or bread consumption increased to 5 and 10%, respectively. Furthermore, the proportion categorized accordingly into the lowest quintile of total whole grain and fiber intake or bread consumption decreased from 65 to 37% for fiber and from 59 to 46% for bread. The changes were equivalent for those in the lowest total whole grain intake quintiles and the lowest two fiber intake or bread consumption quintiles. The proportions categorized into the same or adjacent quintile remained nearly identical after the exclusion of energy under-reporters. When the intakes were examined as g/MJ, the results were similar, except for the effect of excluding energy under-reporters appearing smaller for the correspondence of total whole grain intake with dietary fiber intake and bread consumption (data not shown).

**Table 4:** Cross-classification between quintiles of total whole grain intake (g/d) and whole grain food-definition-based whole grain intake and intake of five potential whole grain surrogate estimates in total population and excluding energy under-reporters

	Lowest total whole grain intake quintile			Proportion categorized in the same quintile (%)	Proportion categorized in the same or adjacent quintile (%)
	Lowest intake quintile (%)	Lowest two intake quintiles (%)	Highest intake quintile (%)		
Whole grain (whole grain food definition^a^					
Total	92	100	0	89	100
Excluding energy under-reporters^b^	93	100	0	88	100
Dietary fiber					
Total	65	86	2	47	88
Excluding energy under-reporters^b^	37	67	5	47	88
Bread					
Total	59	86	4	49	87
Excluding energy under-reporters^b^	46	72	10	48	86
Rye bread					
Total	59	96	0	48	93
Excluding energy under-reporters^b^	66	97	0	50	93
Rye, oat and barley combined					
Total	92	100	0	88	100
Excluding energy under-reporters^b^	91	99	0	87	100
Rye					
Total	66	98	0	52	96
Excluding energy under-reporters^b^	69	99	0	54	96

^a^Whole grain intake from foods with ≥ 30% whole grain ingredients and more whole than refined grains on a dry-weight basis [29]

^b^Energy under-reporters were identified as participants with the ratio between energy intake and basal metabolic rate ≤ 1.14 (Goldberg cut-off value) [32, 33]

## Discussion

This is the first study to assess the suitability of five potential whole grain surrogate estimates to measure total whole grain intake in a Western population within the context of epidemiological research. In addition, whole grain intake based on the Healthgrain Forum’s whole grain food definition was examined in relation to total whole grain intake. Among 5094 Finnish adults participating in the national FinHealth 2017 Study, the correspondence was consistently the greatest between total whole grain intake and intakes of whole grain (whole grain food definition) and the combination of rye, oat and barley regarding both correlations and cross-classification of quintiles. Consumption of rye bread and rye corresponded reasonably well with total whole grain intake.

Thus far, observational studies have focused on the accuracy of whole grain intake estimates when whole grain food definitions with cut-off values for whole grain content have been applied. In the UK, applying a 51% cut-off resulted in a significant reduction in the estimated whole grain intake compared to total whole grain intake among 3073 British participants aged ≥ 1.5 years [13]. Another study comparing two cohorts of British adults (*n* = 2086, aged 16–64 years; *n* = 1692, aged 19–64 years) reported that the 51% cut-off underestimated whole grain intake by up to 27% in comparison with a 10% cut-off [19]. With a 25% cut-off, the underestimation was up to 10%. Moreover, when the Healthgrain Forum whole grain food definition was applied in the Australian (*n* = 12 153, aged ≥ 2 years) and Swedish (*n* = 1797, aged 18–80 years) populations, significantly smaller whole grain intakes were detected compared to total whole grain intake [21, 34].

In our study population, whole grain intake was 7–8% lower when the Healthgrain Forum definition was applied compared to total whole grain intake. However, unlike the previous studies, we assessed dietary intake utilizing the FFQ instead of a 24-h recall or a food diary. In our epidemiological context, whole grain food definition-based whole grain intake corresponded extremely well with total whole grain intake, the correlation being 0.99 and the proportion of participants categorized into the same or adjacent quintile in accordance with total whole grain intake being nearly 90%. This is in accordance with our previous findings utilizing 24-h dietary recalls in a subsample of the same study population, suggesting that whole grains are mainly consumed as foods with very high whole grain content, such as rye bread and porridge [35].

Along with the definition-based whole grain intake, all rye-based variables corresponded well with total whole grain intake. The correspondence was especially good regarding the combination of rye, oat and barley, with a correlation of 0.99 and the proportion of participants categorized into the same or adjacent quintile being 100%. Overall, a good correspondence between rye-based variables and total whole grain intake was expected, as in Finland, rye and rye bread are the primary cereal and food sources of whole grains [35]. Moreover, the results regarding the combination of rye, oat and barley were in accordance with our expectations as oat is the second-most important cereal source for whole grains among Finnish adults, and in Finland, both barley and oat are predominantly consumed as whole grain [35].

Of the examined variables, dietary fiber intake and bread consumption had the weakest correspondence with total whole grain intake. This was not surprising, as dietary fiber can originate from numerous other food sources than whole grain cereals in the diet. Furthermore, alongside rye bread, wheat-based bread with low or no whole grain content has been found to cover a significant proportion of bread consumption in our study population [26].

In cross-classification, dietary fiber intake and bread consumption were the most noticeably affected by the exclusion of energy under-reporters in their correspondence with total whole grain intake. The exclusion decreased the proportion of participants categorized accordingly in the lowest and lowest two quintiles and increased the proportion of grossly misclassified. This suggests that the participants within the lowest whole grain intake quintiles under-reported their intake of fiber sources other than whole grain-containing foods. These may include, for example, wheat-based bread and confectionary with no whole grain ingredients. This is supported by the similar but less drastic phenomenon in bread consumption in relation to total whole grain. As the effect of excluding energy under-reporters was the greatest regarding the proportion categorized accordingly in the lowest quintiles, and only minor changes appeared in the overall classification of participants in the same quintiles, energy under-reporting seemed to occur mainly in the lower end of dietary fiber intake, bread consumption and total whole grain intake. As energy under-reporting is a significant challenge in self-reported dietary intake data in most populations, sensitivity to under-reporting should be considered in choosing a suitable surrogate estimate. Indications of the crudeness of dietary fiber as a surrogate estimate for total whole grain intake have also appeared in other populations. For example, in the USA, only 15% of fiber intake was estimated to originate from whole grain-containing foods [36].

In the subgroup analysis, dietary fiber intake and bread consumption appeared to have the biggest between-group differences in the correlations with total whole grain intake. Dietary fiber intake and bread consumption correlated better with total whole grain intake in men than in women and regarding bread consumption, better in participants with obesity than participants with overweight or normal weight. The differences between sexes are in accordance with earlier findings in Finnish adults, suggesting that the contribution of cereals on fiber intake is more pronounced among men, while women get fiber more variedly from different sources [26]. Correspondingly, in men, bread consumption contributes more to total whole grain intake than in women [35]. The stronger correlation in participants with BMI ≥ 30 kg/m^2^ compared to others indicates similarly a more pronounced role of bread as a whole grain source. However, overall, the differences in correlations between sex, age, educational level and BMI groups were small and irrelevant to the interpretation of the results.

The strongest correspondences with total whole grain intake were mediated by rye intake, including especially the combination of rye, oat and barley, as well as rye and rye bread. As high rye consumption is a distinctive characteristic of the traditional Finnish (and some other Nordic) diet, our results may not be applicable to other populations with diverging whole grain sources. Differing whole grain sources also challenge the suitability of whole grain food definitions in the estimation of whole grain intake in different populations. The major whole grain sources within a population define how much the applied cut-off for whole grain content in foods underestimates actual whole grain intake. Thus, even with the same cut-off, the level of underestimation may drastically differ between populations. Estimating whole grain intake based on foods with required whole grain content instead of any amount of whole grains seems to be especially problematic when a large proportion of whole grains are consumed from foods with low whole grain content, as is displayed in the previous studies [13, 19]. Applying such an estimate in epidemiological research might distort the display of existing associations between whole grain intake and health outcomes.

In our study population, applying the Healthgrain Forum definition with a 30% cut-off resulted in only minor reductions in the estimated whole grain intake. Even a stricter cut-off value of 51% would unlikely result in a much greater reduction as only three foods within the FFQ items with small relevance for total whole grain intake would be further excluded from the estimation. Moreover, the participants were predominantly organized into quintiles accordingly between total whole grain intake and definition-based whole grain intake. This suggests that in the Finnish population with a high proportion of total whole grain intake originating from foods with high whole grain content, whole grain intake estimated based on the Healthgrain Forum definition would be a good estimate for an epidemiological study. Furthermore, utilizing the food-level estimate might provide useful insights into whole grains’ health associations while considering the complex interactions between food components. Findings in a few previous studies examining definition-based whole grain intake estimates regarding specific health outcomes have also indicated that the food-based estimate would be suitable for epidemiological research. In a study conducted in the Australian and Swedish populations, applying the Healthgrain Forum definition had only minor effects on the associations between whole grain intake and risk factors of cardiovascular diseases (CVD) [34]. Furthermore, in an American prospective cohort of 42 850 males aged 40–75 years, the association with CVD incidence did not differ whether total whole grain intake or whole grain intake based on a 51% cut-off was used [37].

Overall, further research on potential surrogate estimates of total whole grain intake in different populations is needed. By establishing the suitability of surrogate estimates, research on whole grain intake can be facilitated in situations where total whole grain intake cannot be calculated. Furthermore, standardized use of surrogate estimates validated in the target population promotes the comparability and consistency of the findings and may further consolidate the evidence on whole grains’ health associations. For the use of epidemiological studies, demonstrating the suitability of surrogate estimates regarding specific health outcomes is as well called for. Moreover, further efforts are required to standardize whole grain definitions in research as, along with the inconsistencies in estimation methods, inconsistent definitions limit the comparability between studies and add to the discrepancies in results.

Strengths of this study include a comprehensive evaluation of the correspondence of food, ingredient, and nutrient level variables with total whole grain intake. As the availability of dietary variables differs in research settings, examining the applicability of different level variables facilitates finding a suitable surrogate estimate in varying situations. Furthermore, we conducted the analyses in a large, population-based sample of the Finnish adult population. Dietary intake was assessed by utilizing an FFQ that has been repeatedly validated in the Finnish adult population. Total whole grain intake was estimated by applying a comprehensive, newly developed whole grain database on a dry-weight basis, covering whole grain intake from the whole diet. To consider the possible misreporting of food consumption arising from self-reported dietary intake, we conducted the analyses both in the total population and excluding energy under-reporters.

The limitations of our whole grain intake estimation are linked to the general challenges in utilizing food composition databases. The information on whole grain content in foods originates from different sources, including ingredient labels, information from manufacturers, whole grain content in similar food products and estimated recipes. Thus, the accuracy of the information may vary. Furthermore, due to the constantly expanding and changing supply of industrial food products, the food composition database may not entirely reflect the current food supply at the data-gathering moment. However, these challenges are not unique to our study but concern more widely the application of food composition databases. In utilizing the Healthgrain Forum definition for whole grain intake estimation, we excluded one compliant food within one FFQ item to facilitate the calculation process. This may have affected our findings on the whole grain food definition-based whole grain intake. However, as the weight of the food in question on the FFQ item was very small, it is unlikely that including it in the estimation would have changed the results significantly. Especially as the effect would have been toward even stronger correspondence between total whole grain and definition-based whole grain intake. Finally, as already mentioned, our results cannot necessarily be applied to other populations due to differing characteristics of food consumption and whole grain sources between populations. However, our findings may provide context and a basis for comparison for future studies, furthering the validation of suitable whole grain surrogate estimates in different populations. Moreover, our results strengthen the findings of previous epidemiological studies that have examined whole grain intake in Finnish adults utilizing some of the surrogate estimates we found acceptable.

In conclusion, our study suggests that rye-based variables, especially the combination of rye, oat, and barley, are suitable surrogate estimates for total whole grain intake in the context of epidemiological research in the Finnish adult population. Similarly, whole grain intake based on the Healthgrain Forum whole grain food definition corresponded well with total whole grain intake. Conversely, dietary fiber intake and bread consumption appeared less suitable estimates for total whole grain intake due to their weaker correspondence with total whole grain intake and sensitivity to energy under-reporting. With more harmonized and validated methods to estimate whole grain intake, the evidence on whole grains’ health associations could be consolidated and the efforts to increase whole grain intake in the population strengthened.

## Supplementary Information

Below is the link to the electronic supplementary material.

Supplementary file1 (PDF 117 KB)

## Data Availability

The data set analysed in this study is available upon request through the Findata permit procedure (https://www.findata.fi/en/).
