# Supplementary material for: The relation of whole grain surrogate estimates and food definition to total whole grain intake in the Finnish adult population
Source: Eur J Nutr. 2023 Feb 27;62(4):1821–31. doi: 10.1007/s00394-023-03119-7 (PMC10195720; doi:10.1007/s00394-023-03119-7)
Supplement: Supplementary file 1 — Supplementary file1 (PDF 117 KB) [file 394_2023_3119_MOESM1_ESM.pdf]

Supplementary Information

Title: The relation of whole grain surrogate estimates and food definition to total whole grain intake in the Finnish adult population

Journal: European Journal of Nutrition

Authors: Rilla Tammi, Satu Männistö, Heli Reinivuo, Heli Tapanainen, Jenna Rautanen, Niina E. Kaartinen

Affiliations: Department of Public Health and Welfare, Finnish Institute for Health and Welfare, Helsinki, Finland

Corresponding author: Rilla Tammi, rilla.tammi@thl.fi

This file includes three supplementary tables. Supplementary Table S1 illustrates the contents of the used food frequency questionnaire. Supplementary Table S2 shows the FFQ items that include foods with whole grain ingredients and indicates which items were included in the calculation of whole grain intake based on the Healthgrain Forum whole grain food definition. Supplementary Table S3 shows the correlations between total whole grain intake and whole grain intake surrogate estimates and definition-based whole grain intake in population subgroups when energy underreporters were excluded from the analyses.

Supplementary Table S1. Summary of items included in the Food Frequency Questionnaire (FFQ) and food codes from the Finnish Food Composition Database used to compose the FFQ items.

Food group	FFQ items	Food codes
Milk products	13	44
Cereal products	16	75
Fat spreads	4	12
Vegetables and vegetable dishes	20	66
Potato, pasta, and rice	6	26
Meat and meat dishes	18	68
Fish and fish dishes	9	32
Poultry dishes and eggs	3	11
Fruits and berries	10	30
Desserts	4	13
Confectionary and snacks	7	25
Beverages	24	49
Total	134	451

Supplementary Table S2. The Food Frequency Questionnaire (FFQ) items that include whole grain containing foods and foods compliant with the Healthgrain Forum whole grain food definition.

FFQ items including whole grain containing foods	FFQ items including foods compliant with the Healthgrain Forum whole grain food definition[a]
Rye bread	X
Multigrain bread, graham roll or toast	(X)[b]
Crisp bread or rye crisp	X
Gluten free bread or bakery product	X
Porridge or gruel	X
Breakfast cereal or Weetabix	X
Muesli	X
Karelian pasty[c]	
Savoury pie or pasty	
Cake, muffin, swiss roll or pastry	
Biscuits	
Pasta, macaroni casserole or lasagne	X
Liver or blood-based dishes	X
Baltic herring[d]	
Pikeperch, whitefish, perch, vendace or pike[d]	
Potato chips and popcorn	X

[a]Foods with ≥30% whole grain ingredients and more whole than refined grains on a dry-weight basis [29].
[b]One food within Multigrain bread, graham roll or toast-item was compliant with the Healthgrain Forum definition but was excluded from the whole grain intake calculation due to its low weight on the item and to facilitate the calculation process.
[c]Traditional Finnish pasty with rye crust and boiled rice.
[d]Whole grain flour in breading.

Supplementary Table S3. Correlation of daily total whole grain intake with whole grain food definition-based whole grain intake and intake of five potential whole grain surrogate estimates by sex, age, education and body mass index (BMI), excluding energy under-reporters.

	Total whole grain (g)											
	Women	Men		Age <58[a]	Age ≥58[a]		Education medium/low[b]	Education high[b]		BMI <30 kg/m²	BMI ≥30 kg/m²	
Dietary intake (g/d)	r[c,d]		*P*[e]	r[c,d]		*P*[e]	r[c,d]		*P*[e]	r[c,d]		*P*[e]
Whole grain (whole grain food definition)[f]	0.99	0.99	0.88	0.99	0.99	0.93	0.99	0.99	0.20	0.99	0.99	0.62
Dietary fiber	0.63	0.75	<0.0001	0.66	0.63	0.24	0.69	0.66	0.11	0.67	0.66	0.63
Bread	0.63	0.74	<0.0001	0.68	0.65	0.15	0.69	0.66	0.13	0.66	0.73	0.0043
Rye bread	0.86	0.84	0.22	0.85	0.83	0.26	0.84	0.86	0.15	0.85	0.85	0.60
Rye, oat and barley combined	0.99	0.99	<0.0001	0.99	0.99	0.35	0.99	0.99	0.61	0.99	0.99	0.18
Rye	0.87	0.86	0.27	0.86	0.85	0.25	0.86	0.87	0.21	0.86	0.87	0.41

[a]Categorized according to the median age.
[b]Participants were classified into educational tertiles based on self-reported total number of school years and by sex and birth year to consider the extension of the basic education system and increase in school years over time.
[c]Partial Spearman's rank correlation coefficient controlled for energy intake.
[d]Energy under-reporters were identified as participants with the ratio between energy intake and basal metabolic rate ≤1.14 (Goldberg cut-off value) [32,33].
[e]Differences between independent sample correlation coefficients were tested using Fisher Z scores.
[f]Whole grain intake from foods with ≥30% whole grain ingredients and more whole than refined grains on a dry-weight basis [29].
